# Preliminary Study on the Preparation of Conductive Nanosized Calcium Carbonate Utilizing Biogas Slurry by a Synchronous Double Decomposition Coating Method

**DOI:** 10.3390/nano13131938

**Published:** 2023-06-26

**Authors:** Fanghui Pan, Han Xiao, Fei Huang, Hongguang Zhu, Jingjing Lei, Jie Ma

**Affiliations:** 1 College of Mechanical and Energy Engineering, Tongji University, Shanghai 201804, China; pfh20072008@126.com (F.P.); 2030252@tongji.edu.cn (H.X.); 2 Modern Agricultural Science & Engineering, Institute of Biomass Energy Research Centre, Tongji University, Shanghai 201804, China; hf173810@163.com; 3 School of Ecology and Environment, Anhui Normal University, Wuhu 241000, China; 4 Research Center for Environmental Functional Materials, College of Environmental Science and Engineering, Tongji University, Shanghai 200092, China; jma@tongji.edu.cn

**Keywords:** biogas slurry, synchronous double decomposition method, humic-acid-based nanosized calcium carbonate, conductive nanosized calcium carbonate

## Abstract

Nanosized calcium carbonate (NCC) plays a vital role in the rubber and plastic fields as a filler, but it cannot resolve the electrostatic problem. Humic-acid-based NCC (HA-NCC) was accidentally discovered in the reaction between biogas slurry and calcium chloride (CaCl_2_), based on nutrient recovery and gradient treatment technology to solve the biogas slurry problem. A preliminary study on the preparation of conductive nanosized calcium carbonate (CNCC) from the HA-NCC was implemented. Meanwhile, a synchronous double decomposition coating method was proposed to properly explain the formation of HA-NCC in the biogas slurry. The CNCC was further obtained through drying and carbonizing the HA-NCC sample. The morphology of CNCC was a square shape with aggregation, and its crystals were calcite. The C content of CNCC was 5% higher than that of the normal CaCO_3_, implying a synchronous coating effect of soluble HA in biogas slurry on NCC. The weight loss of CNCC was about 2.5% at 630 °C, explaining why the HA-NCC remained black at 550 °C for 4 h. The CNCC was partly ordered and graphitized. The resistivity of the CNCC reached 2.62 × 10^6^ Ω·cm. It could be used as a conductive powder. In view of the favorable characteristics described above, CNCC would be expected to be a filler and antistatic agent for plastics and rubbers to enhance the tensile and bending resistance of polymer materials, while eliminating electrostatic hazards. The results are also of great significance for developing high-end products to realize resource utilization of biogas slurry.

## 1. Introduction

NCC is usually from 10 to 100 nm in size, with a large specific surface area and high surface energy [1,2,3]. It has three basic crystal types: calcite, aragonite, and vaterite [4,5]. Calcite is the most thermodynamically stable anhydrous form [6,7]. The nanometric effects and some special properties of NCC have attracted considerable attention not only as an important building material in organisms, but also as a widely used filler in plastics, rubber, paints, coatings, paper, pharmaceuticals, and agrochemicals [8,9,10]. Importantly, appropriate addition of NCC into plastics and rubbers could enhance the toughness, tensile strength, and thermal stability of the products [11,12,13]. However, plastic or rubber, as a kind of insulator (such as polyvinyl chloride (PVC)) with resistance higher than 10^15^ Ω [14,15], has the characteristics of triboelectric charge, and the surface charges can be retained for a long time [16], causing static charge accumulation and electrostatic hazards [17,18]. In order to eliminate the electrostatic problems of polymer materials, conductive powder, as an antistatic agent, could be added to plastics or rubbers to decrease their resistivity [19,20]. For instance, graphene/NCC/PVC composite resins were prepared using graphene and NCC as fillers through an in situ polymerization process, to enhance the thermal stability and prevent electrostatic discharge of PVC [21,22,23]. However, the composite resins have found it difficult to overcome the agglomeration of graphene to date.

NCC without conductivity cannot resolve electrostatic issues as a filler of plastic or rubber. If NCC formed a conductive powder via coating of an inorganic or organic conductive material, it would be not only a filler but also an antistatic agent [22,24]. CNCC is a kind of composite nanomaterial with both filler and antistatic functions, which is different from the traditional conductive powders of the metal, metal oxide, and carbon series [21,25]. Compared with the combined addition of NCC and conductive powders to polymer materials [11,22], CNCC could bestow the polymer materials of plastic and rubber with antistatic properties to prevent electrostatic damage. Meanwhile, the excellent performance of NCC itself could enhance the toughness, tensile strength, and thermal stability of plastic or rubber [11,12]. Moreover, CNCC, with its merits of superfine particle size, large amounts, and comfortable interface compatibility, could overcome ordinary antistatic agents with large particle size, long inter-particle distance, and poor dispersion and interface compatibility, leading to poor conductivity, no antistatic effect, reduced safety, and antistatic failure for common antistatic agents [14,20].

A special preparation method has to be taken into consideration to obtain CNCC. It is understood that CNCC is a composite conductive powder coated with inorganic or organic conductive materials using NCC as a substrate via an asynchronous method [26]. The shortcomings of the asynchronous method are its complex process, high cost, and obvious agglomeration, triggering an increase in particle size and affecting the quality and uniformity of the conductive powders. NCC production is synchronized with conductive material coating, which could effectively control the particle size growth of inorganic nanoparticles and avoid aggregation of nanoparticles to resolve the issues of uneven dispersion and easy agglomeration of nanopowders in inorganic/organic composites [27,28]. Shen et al. [29] used sodium carbonate (Na_2_CO_3_) and CaCl_2_ in a mixed solution of polyethylene pyrrolidone (PVP) and sodium dodecylbenzene sulfonate (SDBS) to prepare NCC by the metathesis method and ultimately obtained spherical NCC with adequate dispersion. However, the double decomposition reaction and synchronous coating process relied on expensive chemical reactants, limiting the application of the process for staple products of NCC.

Hence, the cost-effective carbonates and organic coating materials have been the key point for producing CNCC using a simultaneous double decomposition coating method. Industrial biogas slurry was used to produce CNCC by a synchronous double decomposition coating method in this study. A solution of CaCl_2_ was first added to the biogas slurry to cause the flocculent to precipitate based on bicarbonate stoichiometry. Then, the flocculent precipitate was separated, dried, and carbonized to obtain CNCC. The characteristics of the CNCC were further examined and analyzed. Biogas slurry is the product of anaerobic fermentation of organic waste [30,31]. Due to the use of anaerobic fermentation to achieve the hydrolytic transformation of organic matter, biogas slurry contains high concentrations of ammonia nitrogen, bicarbonate ions, and soluble humic acid. These chemical components of biogas slurry could replace the cost-effective carbonates and organic materials to achieve macroscale preparation of CNCC via the synchronous double decomposition coating method. It is of great significance to implement resource utilization of biogas slurry and develop high-end CNCC products to resolve the electrostatic issues in the plastic and rubber fields.

## 2. Materials and Methods

### 2.1. Raw Materials and Chemical Reagents

The wet biogas slurry treated in the experiment was derived from the effluent of the anaerobic fermentation tank at the Miaoji Biogas Station (32°38′7.68″ N, 115°43′3.24″ E), in Funan County, Fuyang City, Anhui Province, China, where it was separated into solid and liquid portions by the mixer. During the test period (1 April 2022 to 14 October 2022), the chemical oxygen demand (COD) of the biogas slurry was 4063~5207 mg/L, the ammoniacal nitrogen (NH_4_^+^-N) was 2424~3024 mg/L, and the total alkalinity (in terms of CaCO_3_) of the bicarbonate ions (HCO_3_^−^) was 8759~14,264 mg/L. The hardness (in terms of CaCO_3_) was 70–220 mg/L, the pH was 8.113~8.327, and the water temperature was 14.1~29.4 °C. The methods used to measure COD, NH_4_^+^-N, alkalinity, and hardness were as reported in [32].

CaCl_2_, calcium oxide (CaO), sodium hydroxide (NaOH), Na_2_CO_3_, hydrogen peroxide (H_2_O_2_), anhydrous ethanol, and hydrochloric acid were used as analytical reagents (ARs) during the test.

### 2.2. Preparation of CNCC

The wet sample of humic-acid-based nanosized calcium carbonate (HA-NCC) was obtained by a simultaneous double decomposition coating reaction from biogas slurry, CaCl_2_, and HA. According to the molar alkalinity (HCO_3_^−^) of 1 L of biogas slurry (0.25 mol/L, 12,513 mg/L) in the lab-scale experiment, the amount of CaCl_2_ was calculated (see Equation (1)). Due to excess Cl^−^ ions, the biogas slurry exhibited acidity. Therefore, the molar ratio between CaCl_2_ and HCO_3_^−^ was 0.425. That is, 11.79 g of CaCl_2_ was dissolved with 17 mL of pure water. CaCl_2_ solution was added to 1 L of biogas slurry and stirred slowly on a magnetic agitator for 10 min (MS-500, Shanghai, China), followed by static settlement for 20 min. The precipitates of moist HA-NCC were collected through solid–liquid separation. For more detailed preparation of HA-NCC, see reference [32].

Then, the sample of HA-NCC was dried in the oven (DHG-9030A, Shanghai, China) at 105 °C for 12 h to obtain dry HA-NCC. After drying, the dry sample was put into a circular crucible, which was wrapped with tin foil for oxygen isolation. The sample, together with the crucible, was carbonized in a muffle furnace (3X2-4-10H, Shanghai Jing Qi, Shanghai, China) at 550 °C for 4 h. Finally, the carbonized sample was placed in a drying vessel to cool down to room temperature to obtain CNCC as a black powder.

### 2.3. Characterization Methods

The morphology of black CNCC prepared from biogas slurry was observed by SEM (ZEISS GeminiSEM 300, Jena, Germany). An X-ray diffractometer (XRD) (Rigaku Ultima IV, Takatsuki, Japan) and an X-ray fluorescence spectrograph (XRF) (SHIMADZU XRF-1800, Kyoto, Japan) were used to analyze the phase and elemental compositions of CNCC. The weight changes of CNCC at different temperatures were analyzed by thermogravimetric analysis (TGA) (Mettler STAR, Greifensee, Switzerland). The specific surface area, total pore volume, and pore size of CNCC were determined using a BET instrument (BELSORP MR1, MicrotracBEL, Osaka, Japan). Raman spectrometry (Raman) (Horiba LabRAM HR Evolution, Kyoto, Japan) was used to characterize the structure and properties of CNCC. The CNCC powder was identified by a four-probe instrument (RTS-2, Guangzhou Four Probe Co., Ltd., Guangzhou, China).

## 3. Results

### 3.1. HA-NCC Formed in Biogas Slurry

The theory of synchronous metathesis encapsulation states that an organic polymer is mixed with an inorganic precursor in a solvent so that the inorganic precursor chemically synthesizes nanoparticles and fills into the polymer. The existence of polymers can effectively control the growth of inorganic nanoparticles and prevent the agglomeration of nanoparticles.

**Figure 1:**
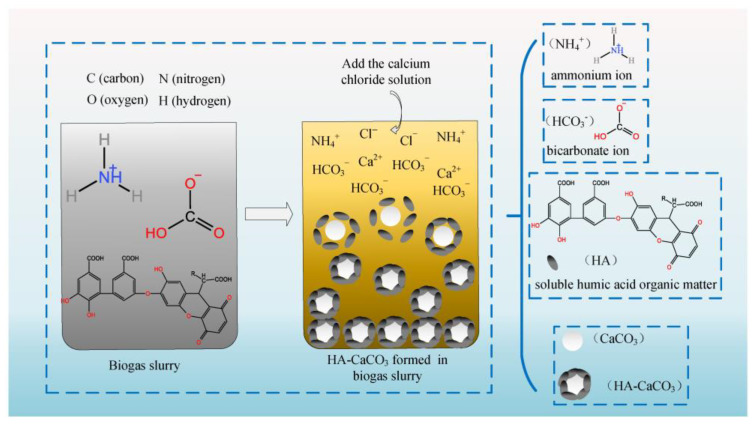
Preparation of humic-acid-based nanosized calcium carbonate (HA-NCC) from biogas slurry by a simultaneous double decomposition coating method.

Biogas slurry is rich in ammonium ions (NH_4_^+^), bicarbonate ions (HCO_3_^−^), and soluble organic matter such as humic acid (HA) [30,31,33]. The content of HA in biogas slurry was measured based on COD concentration. The COD concentration declined from 4018 mg/L to 2649 mg/L. This result indicated that 34% of HA participated in the reaction to produce CaCO_3_ from biogas slurry [32]. The solution of CaCl_2_ was put into the biogas slurry to cause a double decomposition reaction, forming a crystal nucleus of calcium carbonate (CaCO_3_). Due to the enrichment of HA in biogas slurry, it coated the surface of the crystal nucleus of CaCO_3_. Furthermore, it inhibited the infinite growth of the crystal nucleus and was helpful in controlling the particle size of NCC. It also prevented the agglomeration of particles. The HCO_3_^−^ of the biogas slurry and CaCl_2_ solution subjected to the double decomposition reaction to produce CaCO_3_ is presented in Equation (1). The mechanism of forming HA-NCC from biogas slurry through synchronous double decomposition coating is shown in Figure 1.

(1) $${CaCl}_{2}+2{NH}_{4}H{CO}_{3}\to {CaCO}_{3}↓+{CO}_{2}↑+2{NH}_{4}Cl+H_{2}O$$

### 3.2. Morphological Features of CNCC

**Figure 2:**
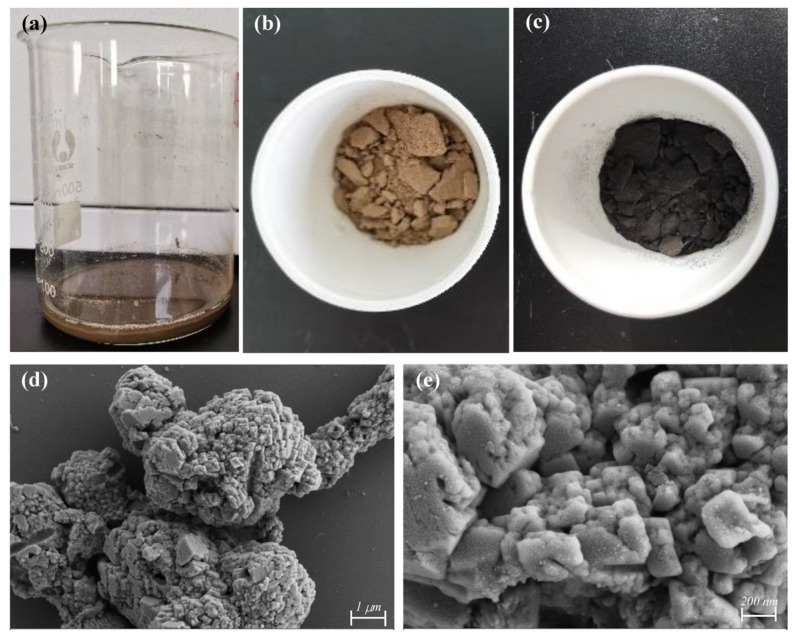
Optical photographs of wet HA-NCC prepared in biogas slurry (**a**), dry HA-NCC (105 °C, 12 h) (**b**), and CNCC heated at 550 °C for 4 h (**c**). SEM images of CNCC at 10,000× magnification (**d**) and 50,000× magnification (**e**).

Figure 2 shows the morphology of CNCC obtained from HA-NCC through the double decomposition coating reaction of CaCl_2_ and biogas slurry, after carbonization for 4 h at 550 °C. The reaction of biogas slurry with CaCl_2_ produced the wet sample of HA-NCC (Figure 2a). The dry HA-NCC after drying at 105 °C for 12 h is displayed in Figure 2b. Figure 2c shows the CNCC sample formed by the carbonization of the dry HA-NCC after oxygen separation at 550 °C for 4 h. According to the SEM observations, the morphology of CNCC showed an encapsulated lamellar shape at 10,000× (Figure 2d), with the agglomeration of irregular and uneven calcite blocks at 50,000× (Figure 2e). This result illustrates that CNCC, with its relatively large particle size, exceeded the nanoscale range with extreme agglomeration (Figure 2d,e). Meanwhile, the NCC was coated by the carbon layer that originated from soluble organic matter, such as HA from biogas slurry (Figure 2d,e). This might be related to the high surface activation energy of CaCO_3_ and the formation of a carbon skeleton by high-temperature carbonization of soluble organic matter such as HA [34,35]. The results indicate that the CNCC should be further crushed and classified or put into a dispersant to control its particle size within 100 nm [36].

### 3.3. Crystal Structure and Composition of CNCC

**Figure 3:**
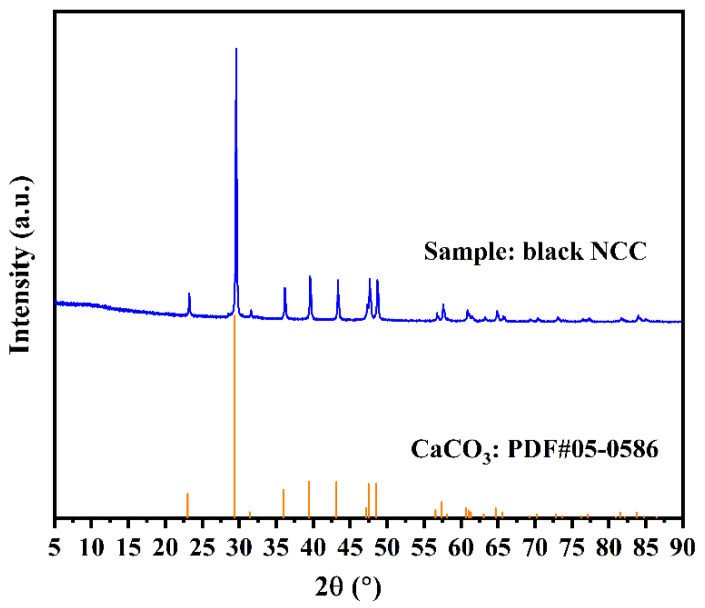
XRD results of CNCC at 550 °C for 4 h.

Figure 3 shows the XRD peaks of CNCC. When CaCO_3_ appeared, the diffraction peaks at 2θ = 23.0°, 29.4°, 36.0°, 39.4°, 43.1°, 47.5°, 48.5°, 56.4°, and 57.5° corresponded to the lattice planes of (012), (104), (110), (113), (202), (018), (116), (211), and (122), respectively. These results indicate that the crystalline form of calcium carbonate was calcite according to the PDF#05-0586 standard, which is consistent with the findings of previous studies [28]. Calcite is more thermodynamically stable than aragonite and vaterite for the crystal structure of CaCO_3_ [7,37]. The XRF elemental composition analysis of CNCC was performed as shown in Table 1. The results showed that the main elements of CNCC were calcium (Ca), oxygen (O), and carbon (C), further supporting the XRD analysis results. The mass fraction of Ca, O, and C in anhydrous CaCO_3_ accounted for 40%, 48%, and 12%, respectively. The XRF analysis results indicated that the C content of the CNCC was 5% higher than that the actual CaCO_3_ content. This part of C might have originated from the coating effect of soluble organic matter such as soluble HA in biogas slurry. Those organic components of biogas slurry—including HA, volatile fatty acids (VFAs), and other biomolecules with chemical characteristics (functional groups)—will be identified and proven in an upcoming study.

### 3.4. TGA and BET

**Figure 4:**
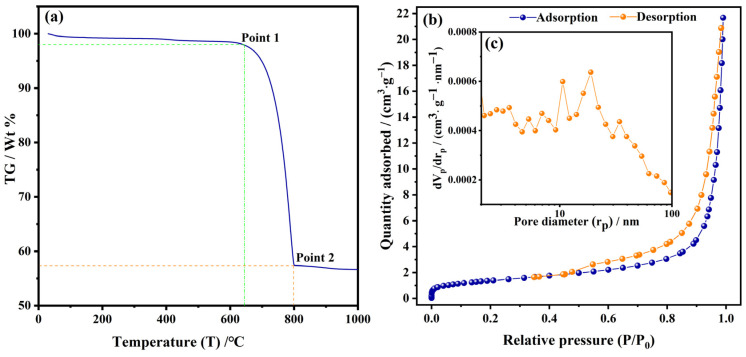
TGA curve (**a**) and N_2_ adsorption–desorption isotherm curve (**b**,**c**) of CNCC.

Figure 4a shows the TGA curve of CNCC. When the temperature rose to approximately 630 °C, the weight loss of the CNCC was about 2.5% (Point 1). This result might be related to the volatilization of the bound water and some part of the organic matter, which explains why the HA-NCC remained black at 550 °C for 4 h. When the temperature was increased to 800 °C, the weight loss of the CNCC was approximately 42.5% (Point 2). This result illustrates that the CNCC was completely decomposed at this high temperature. Its weight loss was about 5% at 700 °C. This might be because the HA coating material was volatilized above 650 °C. The TCA curve of CNCC was a little different from the previous result of the HA-NCC sample in reference [32], and CaCO_3_ was not completely decomposed below 800 °C [2,38]. Figure 4b shows the N_2_ adsorption–desorption isotherm of CNCC. With the increase in the relative pressure, the adsorption capacity increased in the curve. When the relative pressure was P/P_0_ > 0.4, the isotherm showed a hysteresis ring, indicating that the CNCC had mesoporous structure. Figure 4c illustrates the trend of volume distribution with micropores. The BET surface area and total pore volume were 5.03 m^2^·g^−1^ and 0.033 cm^3^·g^−1^, respectively, and the average pore size was 26.33 nm.

### 3.5. Graphitization and Conductivity of CNCC

**Figure 5:**
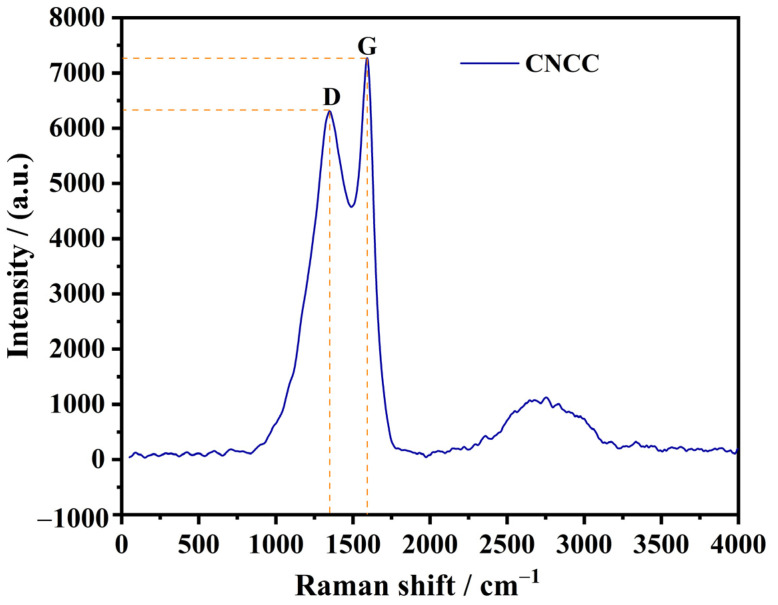
Raman spectra of CNCC. Peak D represents the defect vibration of the carbon atom lattice and peak G signifies the expansion vibration of the sp2 carbon atom.

Figure 5 shows the Raman spectra of CNCC. There were two characteristic peaks at 1350 cm^−1^ and 1590 cm^−1^, which were attributed to the defect vibration of the carbon atom lattice (peak D) and the expansion vibration of the sp2 carbon atom (peak G), respectively. The intensity ratio of peak D and peak G (I_D_/I_G_) represents the degree of graphitization of the carbon materials. The lower the value of I_D_/I_G_, the higher the order and integrity of the carbon material, with a stronger graphite structure [23,27]. The results indicated that CNCC had a certain degree of order and graphitization. The resistivity and conductivity results of CNCC, along with the comparative values of other materials, are shown in Table 2. The resistivity and conductivity of CNCC were 2.62 × 10^6^ Ω·cm and 3.8 × 10^−7^ S·cm^−1^, respectively. The resistivity of CNCC was lower than that of CaCO_3_ and silicon dioxide (SiO_2_) coated with CaCO_3_, and higher than that of polyaniline (PANI) and polyaniline silicon dioxide (SiO_2_-PANI) coated with CaCO_3_ as a conductive powder. However, the resistivity of CNCC was lower than that of nickel (Ni) coated with CaCO_3_, under the same mass ratio of CaCO_3_ and Ni. It has been reported that the resistivity of commercially available antistatic agents and conductive powders ranges from 10^−5^ to 10^9^ Ω·cm [15]. The resistivity of CNCC was within the required range for conductive powders. This result implies that the black and graphitized CNCC obtained from the heat-treated samples at 550 °C for 4 h has significant potential to improve the electrically conductive properties of rubber as a filler. 

## 4. Discussion

### 4.1. A Meaningful Discovery in Biogas Slurry

The experiment was designed to use a strong electrolyte (chloride ions (Cl^−^) derived from CaCl_2_) to replace the weak electrolyte HCO_3_^−^. The original intention of the experiment was to solve the problem of low conductivity and high energy consumption caused by the weak electrolysis of HCO_3_^−^, because the weak electrolysis not only affects electrodialysis (ED) for the recovery of ammonium ions (NH_4_^+^) and potassium ions (K^+^), but also accelerates the fouling and scaling of ED due to CaCO_3_ generation from the chemical reactions between HCO_3_^−^, OH^−^, and Ca^2+^ from biogas slurry. The result was an exciting and surprising flocculation precipitation phenomenon, which was designated as HA-NCC by the research team. When the flocculated precipitates were dried and carbonized, they turned black at 550 °C. The physical, chemical, and electrical properties of the CNCC were first investigated. Currently, biogas slurry nutrient recovery and graded gradient treatment engineering, at a full scale of 200 t/d, is carried out in Tianji Biogas Station (32°44′5.46″ N, 115°38′16.67″ E), Funan County, Fuyang City, Anhui Province, China. Here, 4.15~5.24 t/d of HA-NCC was produced through the engineering to solve the weak electrolyte of HCO_3_^−^ in biogas slurry to mitigate membrane fouling in electrodialysis for nutrient recovery from biogas slurry. In order to achieve the resource utilization of biogas slurry, the macroscale preparation of HA-NCC should be used to develop high-end functional materials such as CNCC, which could be of economic value to balance the cost of biogas slurry treatment.

### 4.2. Synchronous Double Decomposition Coating Method

The industrial preparation of NCC usually uses carbonization methods. The main limestone was calcined to produce quicklime (Ca(OH)_2_), and carbon dioxide (CO_2_) was further carbonized to produce light CaCO_3_ [4,5]. The influence of Ca(OH)_2_ concentration, CO_2_ flow rate, and temperature on the carbonization process [43] caused high industrial production costs, high energy wastage, and high equipment requirements [38]. The double decomposition method to produce CaCO_3_ was the result of the reaction between the soluble CaCl_2_ liquid and the soluble carbonate solution [36]. Compared with the carbonization method, the double decomposition method has the virtues of a fast reaction rate and high efficiency. Moreover, it is a liquid–liquid reaction with controllable reaction conditions, and it is easy to study its reaction kinetics law [44].

The NCC was formed by the double decomposition reaction of CaCl_2_ solution and HCO_3_^−^ in biogas slurry. Meanwhile, the NCC was coated with dissolved organic matter (DOM) such as HA in the biogas slurry. That is, the HA-NCC in the biogas slurry was formed via the synchronous double decomposition coating method (Figure 1 and Figure 2). The soluble organic matter of HA, with carboxyl, hydroxyl, and other groups, was uniformly dispersed in the biogas slurry [45]. The Ca atoms in the surface molecules of NCC readily chelated with the O atoms in the -OH and -COOH chains of HA [35]. This result was similar to the findings of previous studies on the mechanism of HA regulating the morphology and specific surface area of CaCO_3_ in the carbonization process [34,35]. More importantly, CaCO_3_ microcrystals were coated with soluble HA from biogas slurry, with strong molecular binding ability, overcoming the shortcomings of the asynchronous coating method described below.

It is understood that most CNCC composite conductive powders are prepared by using NCC products coated with inorganic or organic conductive materials as a template [39,42,46]. This preparation of CNCC is called the asynchronous method. The asynchronous method has the defects of complex process, high cost, and obvious agglomeration during the coating process, triggering an increase in particle size and affecting the quality and uniformity of the conductive powders [22,40,46]. However, when using the synchronous double decomposition coating method to produce NCC in an inorganic precursor solvent, the polymer coating can effectively control particle size growth of inorganic nanoparticles and avoid aggregation of nanoparticles, resolving the issues of uneven dispersion in inorganic/organic composites [27,29]. For instance, potassium humate 3D graphene was prepared by adding soluble additives to prevent potassium humate agglomeration [27]. Spherical NCC with a good dispersion was obtained by the reaction of Na_2_CO_3_ and CaCl_2_ in the mixed aqueous solution of polyethylene pyrrolidone (PVP) and sodium dodecylbenzene sulfonate [29]. It is worth noting that the use of cheap carbonates and organic materials has been a vital point in the macroscale preparation of CNCC using the double decomposition coating method. A great deal of CNCC could be prepared by the double decomposition coating method in biogas slurry. It is of great significance to realize the resource utilization of biogas slurry and develop high-end green products.

### 4.3. CNCC

Recently, researchers have been interested in the production of composite conductive powders from NCC and organic materials, because organic conductive materials have stronger corrosion resistance and lower cost than inorganic metal materials when used as coating materials for NCC. Commonly used polymer materials include polyaniline (PANI), polypyrrole (PPy), polyacetylene (PA), and polythiophene (PTi) [22,40]. Among them, PANI has excellent conductivity, antisepsis, and non-toxicity [40]. The conductive powder prepared by PANI coated with NCC not only had good conductivity but also enhanced the thermal stability, mechanical strength, and adhesion of the conductive powder, through synergistic action with NCC.

This study is the first to report the preparation of CNCC from the HA-NCC via synchronous double decomposition coating in biogas slurry, with a resistivity of 2.62 × 10^6^ Ω·cm, adhering to the resistivity requirements of conductive powders (10^−5^~10^9^ Ω·cm) [15,22]. However, it is important to emphasize that the CNCC in this study should be further optimized to reduce its resistivity compared to the other conductive powders listed in Table 2. In summary, CNCC as a filler and antistatic agent could improve the structural function of polymer materials such as plastic and rubber, while also resolving the electrostatic problem. The conductivity of CNCC should be further investigated in upcoming research when CNCC is added to rubber or plastic as a filler.

### 4.4. Limitations and Development Prospects

The CNCC derived from HA-NCC by the synchronous double decomposition coating method, using biogas slurry as a raw material, was used to develop high-end and functional products conforming to market requirements. For one thing, the biogas slurry needed no longer be regarded as sewage and achieved the goal of resource utilization. For another, the formed CNCC powder, as a filler and antistatic agent, improved the structural properties of polymer materials such as plastic and rubber, while also resolving the electrostatic problem. This would be beneficial for the green and high-quality development of enterprises. However, this was only a preliminary study aiming at the preparation of CNCC from the perspective of biogas slurry resource utilization. The CNCC particle size was still large, with a significant agglomeration phenomenon. Future work concerning CNCC should be carried out with regard to the tunable conditions of particle size, specific surface area, and microstructure, as well as electrochemical performance optimization to decrease its particle size, enhance its specific surface area, and improve its conductivity.

## 5. Conclusions

Based on biogas slurry obtained from anaerobic fermentation in a biogas station, using nutrient recovery and graded gradient treatment technology, the use of a strong Cl^−^ electrolyte from CaCl_2_ solution to replace the weak HCO_3_^−^ electrolyte in biogas slurry was investigated. The flocculent precipitates of HA-NCC were discovered unexpectedly, by the reaction of CaCl_2_ and biogas slurry. According to the discovery, the double decomposition coating method was proposed to prepare the HA-NCC. Then, the preparation of CNCC was further preliminarily explored by the HA-NCC. The morphology, structure, composition, specific surface area, and resistivity of the CNCC were also examined primitively. The morphology of CNCC exhibited aggregation consisting of an ample cuboidal matrix. CNCC showed a calcite crystal structure, and its C mass fraction was 5% higher than the C mass fraction of actual CaCO_3_, revealing the coating effect of HA in biogas slurry on the NCC. The TGA results reveal why HA-NCC showed a black color after treatment at 550 °C for 4 h. About 5% of its weight at 700 °C was lost, resulting from the coated C being volatilized in gas form at that temperature. The CNCC had a specific surface area of 5.03 m^2^·g^−1^, total pore volume of 0.033 cm^3^·g^−1^, and average pore size of 26.33 nm, as well as mesoporous properties. The sample was graphitized to an extensive degree. The resistivity of the sample was 2.62 × 10^6^ Ω·cm, implying its conductivity. The CNCC showed the potential to develop a conductive powder. It would be expected to be used as a filler in the plastics and rubber industries, and as an antistatic agent to eliminate electrostatic hazards.

## Figures and Tables

**Table 1:** XRF analysis results of CNCC (550 °C, 4 h).

Chemical Element	Mass Percentage of Chemical Element (%)	Oxide Component	Mass Percentage of Oxide Component (%)
Ca	57.45	CaO	50.35
O	22.01	CO_2_	47.09
C	17.97	P_2_O_5_	0.95
Cl	0.84	Cl	0.61
P	0.56	K_2_O	0.29
Sr	0.36	SrO	0.21
K	0.34	SO_3_	0.15
Na	0.12	Na_2_O	0.12
Zn	0.12	ZnO	0.07
Fe	0.09	SiO_2_	0.07
S	0.08	Fe_2_O_3_	0.06
Si	0.04	Al_2_O_3_	0.03
Al	0.02	-	-
Total	100.00	Total	100.00

**Table 2:** Resistivity and conductivity of CNCC compared with other materials.

Functional Materials	Resistivity/Ω·cm	Conductivity/S·cm^−1^	References
CNCC	2.62 × 10^6^	3.8 × 10^−7^	This study
CaCO_3_	>10^7^	<10^−7^	[39]
CaCO_3_-SiO_2_	>10^7^	<10^−7^	[39]
Polymers	10^15^~10^17^	10^−15^~10^−17^	[15]
Pure polyaniline (PANI)	2.63 × 10^2^	3.8 × 10^−1^	[40]
CaCO_3_-SiO_2_-PANI (1:1~3:1)	1.79 × 10^3^~1.75 × 10^4^	5.6 × 10^−2^~5.7 × 10^−3^	[41]
PANI/CaCO_3_ composites	1.0 × 10^5^	1.0 × 10^−5^	[26]
CaCO_3_-Ni (0.47:1~1:1)	1.2 × 10^6^~3.4 × 10^11^	8.3 × 10^−7^~2.9 × 10^−12^	[42]

## Data Availability

Where no new data were created.
